# Influence of Zinc and Manganese Nanoparticles on Selected Parameters of Turkey Spermatozoa Stored in a Liquid State at 4 °C

**DOI:** 10.3390/ani11113289

**Published:** 2021-11-17

**Authors:** Aleksandra Orzołek, Katarzyna T. Rafalska, Wiktoria A. Otowska, Władysław Kordan, Anna J. Korzekwa, Krzysztof Kozłowski

**Affiliations:** 1 Department of Animal Biochemistry and Biotechnology, University of Warmia and Mazury in Olsztyn, Oczapowskiego 5, 10-719 Olsztyn, Poland; katarzyna.rafalska@uwm.edu.pl (K.T.R.); wiktoria.otowska@gmail.com (W.A.O.); wladyslaw.kordan@uwm.edu.pl (W.K.); 2 Department of Biodiversity of Protection, Institute of Animal Reproduction and Food Research of the Polish Academy of Sciences, Tuwima 10, 10-748 Olsztyn, Poland; a.korzekwa@pan.olsztyn.pl; 3 Department of Poultry Science and Apiculture, University of Warmia and Mazury in Olsztyn, Oczapowskiego 5, 10-719 Olsztyn, Poland; kristof@uwm.edu.pl

**Keywords:** nanoparticles, zinc, manganese, turkey, spermatozoa

## Abstract

**Simple Summary:**

Nanoparticles (NPs) are not widely used in semen preservation procedures due to their potential toxicity as well as limited knowledge regarding safe NP doses. Turkey sperm loses its fertilizing potential when it is stored for more than several hours. Recent years have witnessed an upsurge of interest in novel procedures for improving the storage of turkey semen. The addition of zinc to the extender may improve progressive sperm motility, viability, membrane integrity and DNA stability. The viability, membrane integrity, motility and osmotic balance of sperm can also be enhanced through the addition of manganese, which exerts antioxidant effects. Despite the fact that the influence of NPs on sperm is not fully understood, their biological effects can be harnessed to enhance the quality of sperm cells during semen preservation.

**Abstract:**

The aim of this study was to determine the effect of semen extender supplementation with 25 or 50 μM of zinc nanoparticles (ZnNPs) or manganese nanoparticles (MnNPs) on turkey spermatozoa preserved in a liquid state. Twenty turkey ejaculates were obtained from twenty healthy males. The collected semen was preserved at 4 °C for 48 h with or without NPs. Selected qualitative and quantitative parameters of sperm (motility, plasma membrane activity, mitochondrial membrane potential (MMP) and the percentage of sperm demonstrating NO and SOD activity) were examined after 2, 24 and 48 h of storage. Sperm motility and MMP decreased in semen preserved with ZnNPs at each time point of the analysis. However, all spermatozoa remained viable throughout storage. In contrast, membrane integrity and mitochondria activity (*p* ≤ 0.05) increased, and the highest SOD activity (*p* ≤ 0.05) was observed in semen preserved with MnNPs. The addition of MnNPs to the semen extender could potentially improve the parameters of turkey semen during prolonged storage.

## 1. Introduction

The last two decades have witnessed a growing body of research into the effects of nanoparticles (NPs) on semen parameters. Nanoparticles are molecules with a size of 1 to 100 nm. Due to their unique properties, NPs have attracted considerable interest in biotechnology, medicine, animal reproduction and the veterinary sciences. Nanoparticles have a wide range of practical applications: they are used in semen nano-purification and as nano-protectant additives in semen preservation. Nanoparticles exert similar effects to antibiotics: they protect sperm against bacteria, and improve sperm motility and viability in a dose-dependent manner [1].

Mammalian seminal plasma and spermatozoa contain numerous macroelements and microelements. Zinc (Zn) is necessary for growth; development; sexual maturation; reproduction; dark vision adaptation; olfactory and gustatory activity; and insulin storage and release, as well as host immune responses. Zinc is the only metal to be found in all enzyme classes [2]. It plays an important role in sperm viability and motility. Zinc affects protein metabolism, nucleic acid synthesis and the stabilization of the sperm membrane. This microelement delivers antioxidant effects by preventing defective spermatozoa and leukocytes from producing reactive oxygen species (ROS), inhibiting lipid peroxidation and reducing the levels of circulating anti-sperm antibodies [3]. Zinc participates in sperm capacitation as well as the acrosome reaction, plus it is essential for conception and embryo implantation [4]. Human sperm chromatin contains one zinc ion per each protamine molecule in each turn of the DNA. Therefore, zinc ions probably contribute to the stabilization of sperm chromatin. Low zinc levels in seminal plasma significantly compromise reproductive potential and contribute to the fragmentation of sperm DNA [5].

Manganese (Mn) is essential for controlling blood sugar levels; it offers protection against ROS, and also promotes blood coagulation, nervous system function, bone growth and immune defense. This microelement activates selected enzymes, in particular mitochondrial manganese superoxide dismutase (Mn-SOD), as well as pyruvate carboxylase, glycosyltransferase, glutamine synthetase and alkaline phosphatase [6]. Divalent manganese ions (Mn^2+^) enhance sperm motility, viability, capacitation and the acrosome reaction by minimizing oxidative stress [7,8]. Manganese forms complexes with unsaturated fatty acids and decreases their susceptibility to peroxidation. Furthermore, manganese is far more effective in preventing lipid peroxidation than zinc or nickel [9].

Previous studies have demonstrated that NPs exert antioxidant effects on the semen of selected animal species, including rams, bulls, rats and roosters [10]. The aim of this study was to investigate the influence of zinc and manganese nanoparticles (ZnNPs and MnNPs) on turkey semen preserved in a liquid state. An attempt was made to determine the extent to which the supplementation of a semen extender, with 25 or 50 μM of ZnNPs or MnNPs, affects the parameters of turkey spermatozoa preserved at 4 °C for 48 h. In our previous study (unpublished data), the addition of 100 μM of ZnNPs or MnNPs to stored turkey ejaculates preserved or enhanced their live characteristics, including motility, viability and mitochondrial activity, for at least 2 h. In the present study, efforts were made to determine the optimal and non-toxic concentrations of motility and membrane integrity.

## 2. Materials and Methods

The experimental material consisted of ejaculates (*n* = 20) that were obtained from 20 Big 6 male turkeys (Aviagen, Huntsville, AL, USA), aged approximately 41 weeks. The turkeys were raised on a breeding farm (GERCZAK, Nord-Pol Hatchery, lława, Poland) in Kozia Góra (Warmian-Masurian Voivodeship, Kozia Góra, Poland), where strict hygienic practices and nutritional requirements were observed. Five ejaculates were collected each week by abdominal massage. Every ejaculate was collected with a syringe and immediately diluted with an Extendyl extender (IMV Technologies, L’Aigle, France) in a 1:2 ratio (*v*/*v*). The collected ejaculates were placed in a thermobox (39 °C) and were immediately transported to a laboratory to preserve sperm livability.

### 2.1. Preparation of Nanoparticle Suspensions

ZnNPs and MnNPs were purchased in powder form (SkySpring NanoMaterials Inc., Houston, TX, USA). The size of ZnNPs ranged from 10 to 30 nm, and the size of MnNPs ranged from 40 to 60 nm. Both types of NPs were dissolved to appropriate concentrations (20 and 50 μM) with the use of an Extendyl extender. Fresh turkey ejaculates were diluted with the prepared NP suspensions of 200 × 10^6^ sperm/mL and were preserved at 4 °C for 48 h. One part of each ejaculate was diluted with the extender without the addition of NPs and was used as the control (C).

Semen was preserved for 48 h and selected qualitative and quantitative parameters were examined in fresh semen with or without the addition of NPs after 2, 24 and 48 h of storage.

### 2.2. Determination of Sperm Concentration

Sperm concentration was determined with the use of a light microscope and a Bürker counting chamber (Equimed-Medical Instruments, Kraków, Poland). Each sample was diluted with 0.85% NaCl (to a final ratio of 1:800), then placed in the counting chamber and the spermatozoa were counted under an optical microscope. The total number of spermatozoa was counted in twenty squares of the Bürker chamber, and the average number of sperm cells per square was estimated.

### 2.3. Determination of Sperm Motility in the CASA System

To assess sperm motility, stored sperm samples were diluted to obtain approximately 30 × 10^6^ spermatozoa/mL. The samples were diluted with a motility buffer (50 mM of Tris, 120 mM of NaCl, 10 mM of glucose, 2 mM of CaCl2; pH 7.4) and 0.5% BSA. The prepared samples were heated for around 15 min at a temperature of 39 °C (Thermo Block TDR-120, Germany). Total motility (TMOT, %), progressive motility (PMOT, %), curvilinear line velocity (VCL, μm/s), straight line velocity (VSL, μm/s), average velocity (VAP, μm/s), amplitude of lateral head displacement (ALH, μm), beat cross frequency (BCF, Hz), linearity coefficient (LIN, %) and straightness (STR, %) were measured using a computer-assisted sperm analysis (CASA) system (Hamilton–Thorne Biosciences, IVOS version 12.3, USA) and a Makler chamber. The CASA system was configured as follows: frame acquired–60, frame rate–60 Hz, minimum cell contrast–35, minimum cell size–5 pixels, path velocity (VAP) threshold–50 μ/s, straightness (STR) threshold–80.0%, VAP cutoff–30.0 μ/s, VSL cutoff–15.0 μ/s. All settings were implemented manually according to the Hamilton–Thorne technical guide v.12.3 for roosters.

### 2.4. Determination of Plasma Membrane Integrity (SYBR-14/PI)

The percentage of membrane-intact spermatozoa was assessed with SYBR-14 and propidium iodide (PI) (Live/Dead Sperm Viability Kit, Molecular Probes) fluorescent dyes according to the method proposed by Garner and Johnson [11], with some modifications [12]. Sperm samples were extended to a final concentration of 30 × 10^6^ spermatozoa/mL with an HEPES-buffered saline solution (130 mM of NaCl, 4 mM of KCl, 14 mM of fructose, 10 mM of HEPES, 1 mM of CaCl, 0.5 mM of MgCl, 0.1% BSA) to a volume of 100 μL. The samples were incubated at 39 °C; first, with 1 μL of SYBR-14 solution (1 mM of SYBR-14 in DMSO) for 10 min, and then with 1 μL of PI solution (2.4 µM of PI in Tyrode salt solution) for another 10 min. The incubated subsamples of 10 μL each were placed on sterile microscopic slides and covered with coverslips. A minimum of 200 cells per slide were examined in each aliquot under an epifluorescence microscope (Olympus CH 30 RF-200, Tokyo, Japan). The number of membrane-intact spermatozoa (green fluorescence) and spermatozoa with damaged membranes (red fluorescence) was counted. The results were expressed as the percentage (%) of sperm cells with an intact membrane in the acrosome region.

### 2.5. Determination of Mitochondrial Membrane Potential (JC-1/PI)

Mitochondrial membrane potential (MMP) was assessed with the use of JC-1 (Molecular Probes, Eugene, OR, USA) and PI fluorescent dyes, according to the method described by Thomas et al. [13]. Sperm samples were extended to 30 × 10^6^ spermatozoa/mL with an HEPES-buffered saline solution. The samples were incubated at 39 °C; first, with 1 μL of JC-1 solution for 10 min, and then with 0.3 μL of PI solution (2.4 µM PI in Tyrode salt solution) for another 10 min. The incubated subsamples of 10 μL each were placed on sterile microscopic slides and covered with coverslips. A minimum of 200 cells per slide were examined in each aliquot under an epifluorescence microscope according to the procedure described above. Spermatozoa exhibiting orange fluorescence in the mid-piece region were considered as viable cells with higher MMP (active mitochondria). The results were expressed as the percentage (%) of sperm cells with active mitochondria.

### 2.6. Determination of Nitric Oxide Production

The percentage of sperm cells producing nitric oxide (NO) was determined with DAF-2DA fluorescent dye, according to the method proposed by Lampiao et al. [14]. Sperm samples were diluted to 30 × 10^6^ spermatozoa/mL with an HEPES-buffered saline solution. The samples were incubated at 39 °C with 100 μL of DAF-2DA solution (20 μM DAF-2DA in PBS) for 120 min, in the dark. Aliquots of the obtained suspensions were placed on sterile microscopic slides and covered with coverslips. A minimum of 200 cells per slide were examined in each aliquot under a light microscope and an epifluorescence microscope. Spermatozoa exhibiting blue-green fluorescence in any segment (head, mid-piece or tail) were considered as NO-producing cells. The results were expressed as the percentage (%) of sperm cells in the field of view that were capable of producing NO.

### 2.7. Determination of Superoxide Dismutase Activity

Superoxide dismutase (SOD) activity was measured in sperm extracts with the use of a commercial kit (Randox Laboratories, Crumlin, United Kingdom) in accordance with the instructions of the manufacturer. The test is based on the ability of xanthine and xanthine oxidase to generate superoxide anion radicals. The formed particles react with 2-(4-iodophenyl)-3-(4-nitro-phenol)-5-phenyltetrazolium chloride (INT) to produce formazan (red stain). The evolved color of the solution is directly proportional to SOD activity (measured at 505 nm). One unit of SOD is a quantity that inhibits INT reduction by 50% per minute at 37 °C.

### 2.8. Total Protein Content

The sperm extracts were prepared with the use of Tris-buffered saline (TBS) containing 1% sodium lauryl sulfate (SDS). The total protein content of the sperm extracts was measured according to the method proposed by Bradford [15].

### 2.9. Statistical Analysis

All data were processed statistically with the use of the Statistica program (version 13.1, StatSoft, Poland). The results were presented as arithmetic means ± standard errors of the mean (SEM). Selected sperm parameters were compared by one-way ANOVA with the Newman–Keuls test at a significance level of *p* ≤ 0.05. The presence of correlations between the analyzed parameters was determined by calculating correlation coefficients. The observed correlations were classified as weak (0.2–0.4), moderate (0.41–0.7), strong (0.71–0.9) or very strong (>0.91).

## 3. Results

### 3.1. Sperm Concentration

Sperm concentration was determined in each sample, and it ranged from 5.46 to 2.21 (×10^9^) spermatozoa/mL.

### 3.2. Sperm Motility Analysis in the CASA System

Total sperm motility was determined in each sample. This parameter ranged from 90% to 65% in fresh semen, and motility characteristics did not change after 2 h of storage. After 24 and 48 h of storage, significant differences were noted between samples containing ZnNPs and the remaining samples (Table 1). In samples containing ZnNPs, sperm motility continued to decrease in successive hours of storage. Total motility was significantly lowest (*p* ≤ 0.05) after 48 h of storage in samples preserved with the addition of Zn50NPs (Table 1). In turn, no significant differences in TMOT were observed between samples preserved without NPs (44%), with Mn25NPs (41%) and with Mn50NPs (42%), after 48 h of storage (Table 1).

### 3.3. Plasma Membrane Integrity (SYBR-14/PI)

**Figure 1:**
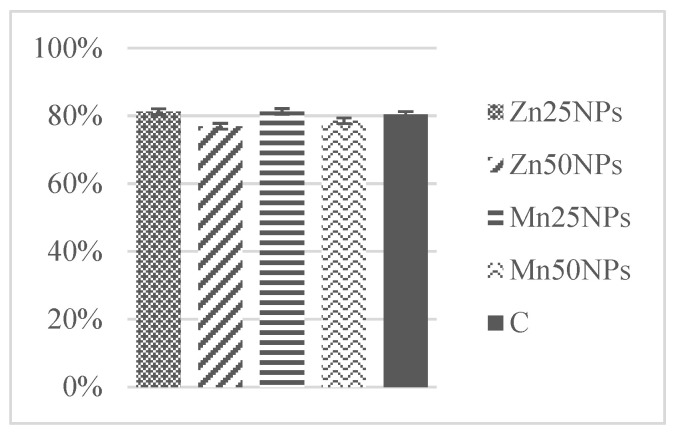
Percentage of sperm with intact plasma membrane (%) in samples stored for 2 h with or without (C) the addition of ZnNPs and MnNPs.

**Figure 2:**
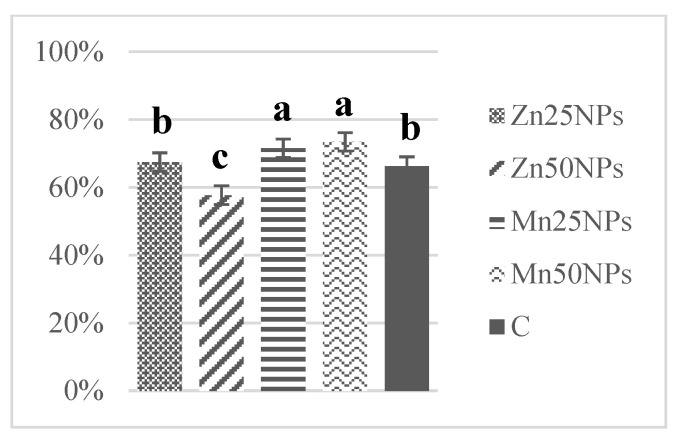
Percentage of sperm with intact plasma membrane (%) in samples stored for 24 h with or without (C) the addition of ZnNPs and MnNPs. * Different letters (a, b, c) indicate statistically significant differences at *p* ≤ 0.05. The bars with various letters are significantly different from one another.

**Figure 3:**
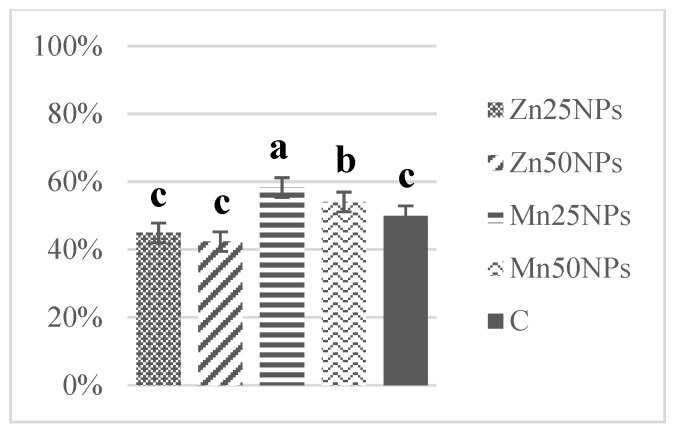
Percentage of sperm with intact plasma membrane (%) in samples stored for 48 h with or without (C) the addition of ZnNPs and MnNPs. * Different letters (a, b, c) indicate statistically significant differences at *p* ≤ 0.05. The bars with various letters are significantly different from one another.

In fresh ejaculates, the percentage of viable spermatozoa with intact membranes ranged from 94% to 74%. After 2 h of storage, the highest numbers of membrane-intact spermatozoa were determined in semen preserved with Zn25NPs and Mn25NPs (Figure 1), but no significant differences were found between the samples. After 24 h of storage, membrane integrity was significantly highest in samples containing Mn25NPs and Mn50NPs (Figure 2). After 48 h of storage, the highest percentage of membrane-intact sperm was noted in samples preserved with Mn25NPs (*p* ≤ 0.05) (Figure 3). In contrast, the number of viable sperm was significantly lowest in samples containing Zn50NPs in each hour of storage (Figure 1, Figure 2 and Figure 3).

### 3.4. Mitochondrial Membrane Potential (JC-1/PI)

**Figure 4:**
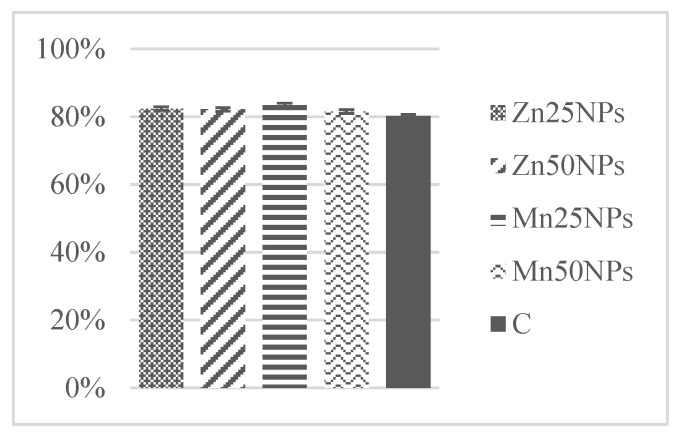
Percentage of turkey sperm with functional mitochondria (%) in samples stored for 2 h with or without (C) the addition of ZnNPs and MnNPs.

**Figure 5:**
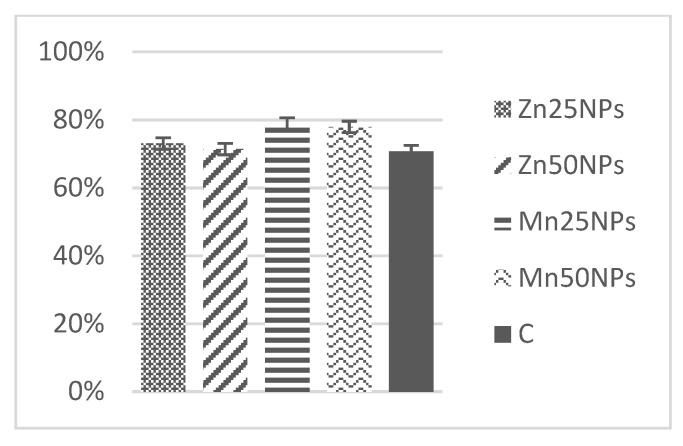
Percentage of turkey sperm with functional mitochondria (%) in samples stored for 24 h with or without (C) the addition of ZnNPs and MnNPs.

**Figure 6:**
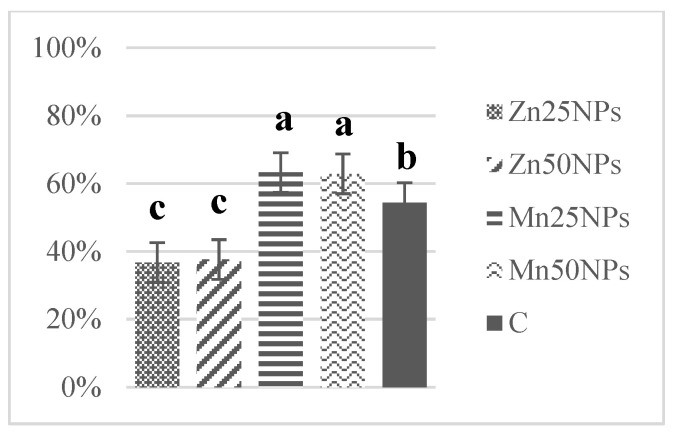
Percentage of turkey sperm with functional mitochondria (%) in samples stored for 48 h with or without (C) the addition of ZnNPs and MnNPs. * Different letters (a, b, c) indicate statistically significant differences at *p* ≤ 0.05. The bars with various letters are significantly different from one another.

The percentage of spermatozoa with high MMP ranged from 91% to 63% in samples of fresh semen. Membrane potential did not change after 2 h of storage (Figure 4). A minor decrease in MMP was observed after 24 h, and a considerable decrease in this parameter was noted after 48 h of storage (Figure 5 and Figure 6). Regardless of storage time, MMP was significantly highest in sperm samples preserved with the addition of MnNPs (Figure 4, Figure 5 and Figure 6). The smallest drop in MMP was observed in semen samples containing Mn50NPs (Figure 6).

### 3.5. Nitric Oxide Production

**Figure 7:**
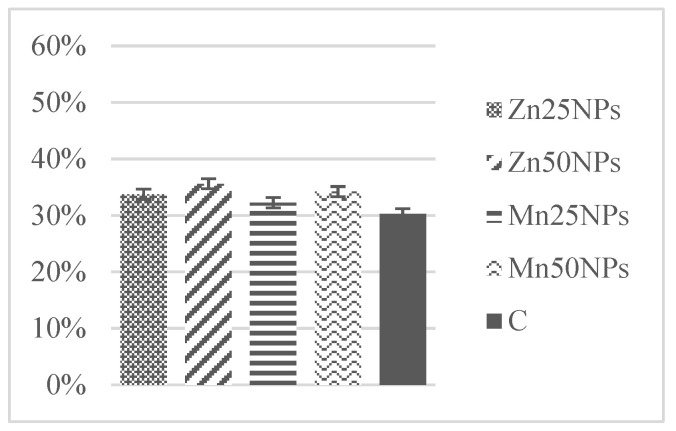
Percentage of turkey sperm producing NO (%) in samples stored for 2 h with or without (C) the addition of ZnNPs and MnNPs.

**Figure 8:**
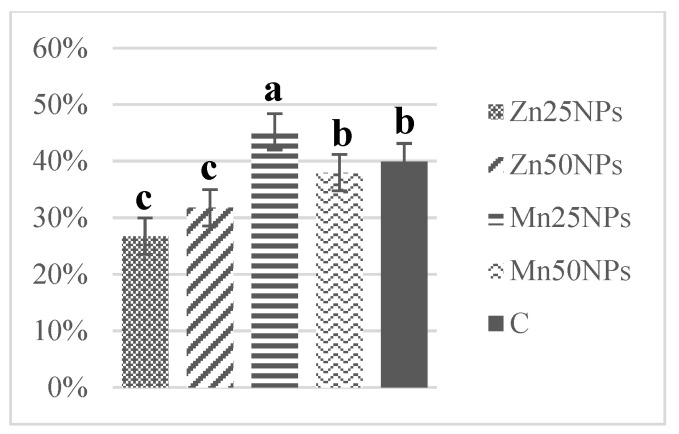
Percentage of turkey sperm producing NO (%) in samples stored for 24 h with or without (C) the addition of ZnNPs and MnNPs. * Different letters (a, b, c) indicate statistically significant differences at *p* ≤ 0.05. The bars with various letters are significantly different from one another.

**Figure 9:**
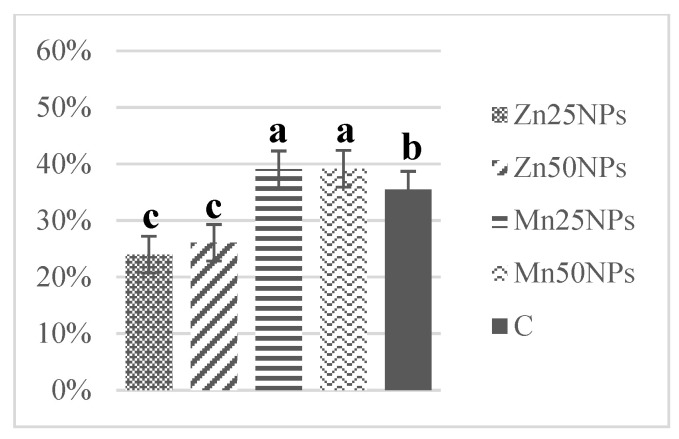
Percentage of turkey sperm producing NO (%) in samples stored for 48 h with or without (C) the addition of ZnNPs and MnNPs. * Different letters (a, b, c) indicate statistically significant differences at *p* ≤ 0.05. The bars with various letters are significantly different from one another.

In fresh semen, the percentage of spermatozoa producing NO ranged from 45% to 5%, and it did not change significantly after 2 h of storage (Figure 7). However, a significant increase in the above parameter was observed in samples preserved with MnNPs after 24 h of storage (Figure 8). In samples containing ZnNPs, the percentage of sperm producing NO had already decreased (*p* ≤ 0.05) on the first day of storage (Figure 8). After 48 h, statistically the highest percentage of spermatozoa exhibiting blue-green fluorescence was noted in samples containing 25 and 50 μM of MnNPs (Figure 9).

**Figure 10:**
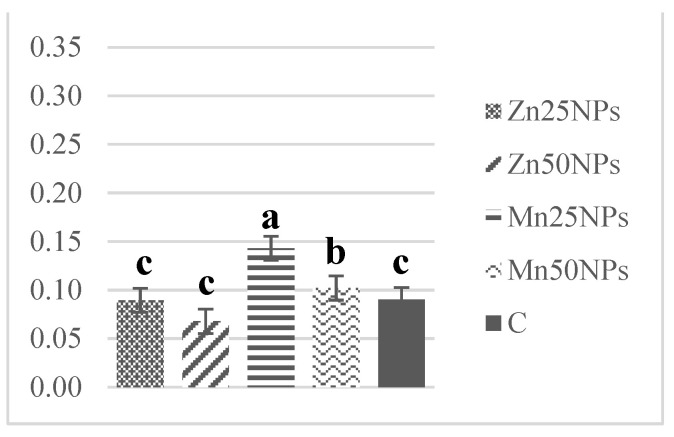
SOD activity (U/mg) in samples of turkey sperm stored for 2 h with or without (C) the addition of ZnNPs and MnNPs. * Different letters (a, b, c) indicate statistically significant differences at *p* ≤ 0.05. The bars with various letters are significantly different from one another.

**Figure 11:**
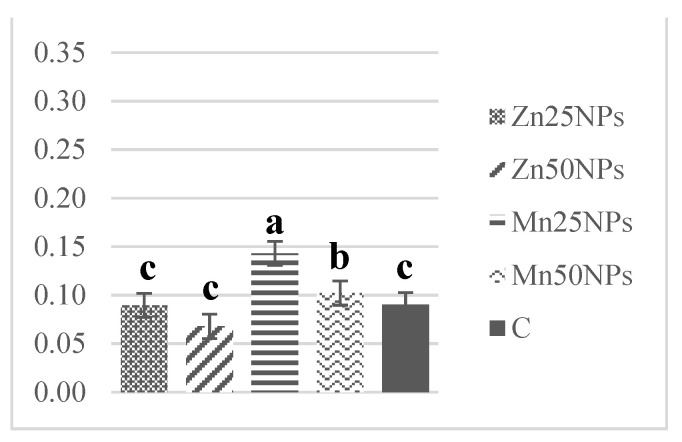
SOD activity (U/mg) in samples of turkey sperm stored for 24 h with or without (C) the addition of ZnNPs and MnNPs. * Different letters (a, b, c) indicate statistically significant differences at *p* ≤ 0.05. The bars with various letters are significantly different from one another.

**Figure 12:**
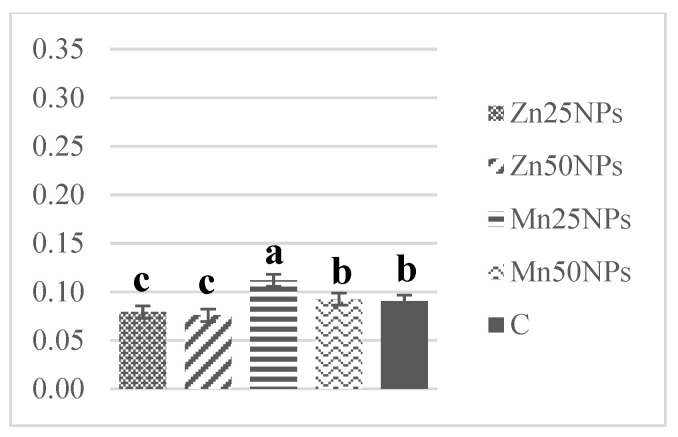
SOD activity (U/mg) in samples of turkey sperm stored for 48 h with or without (C) the addition of ZnNPs and MnNPs. * Different letters (a, b, c) indicate statistically significant differences at *p* ≤ 0.05. The bars with various letters are significantly different from one another.

The SOD content of fresh semen ranged from 1.30 to 0.61 U/mg of protein on average. Superoxide dismutase levels decreased markedly after 2 h of storage, excluding samples preserved with Mn25NPs (Figure 10). A further decline in SOD levels was noted after 24 and 48 h of storage. Superoxide dismutase activity was significantly highest in samples preserved with Mn25NPs at each time point during the study (Figure 10, Figure 11 and Figure 12).

### 3.6. Correlations

In semen preserved with ZnNPs and MnNPs, statistically significant correlations between sperm motility and the percentage of sperm that produced NO was noted as soon as after 2 h of storage. Interestingly, any other parameter was not correlated with sperm motility after this time.

After 24 h of storage, the motility of spermatozoa preserved with nanoparticles was correlated with high MMP (*p* ≤ 0.05) (Table 2). The strongest correlation was observed in sperm containing Mn50NPs. Moreover, positive strong (ZnNPs) and moderate (Mn25NPs) correlations were noted between sperm motility and the percentage of sperm that generated NO. Furthermore, a positive correlation between sperm motility and superoxide dismutase (SOD) activity was demonstrated in samples containing Mn25NPs (Table 2).

The correlations between sperm motility and high MMP were maintained in samples containing Mn25NPs and in control samples after 48 h of storage (Table 2). The strongest correlation was observed in sperm preserved with Mn25NPs. The strongest correlation between motility, plasma membrane integrity and SOD activity, was also noted in samples containing Mn25NPs (*p* ≤ 0.05). Surprisingly, significant correlations between sperm motility and NO production were found only in samples with ZnNP supplementation (Table 2).

After 2 h of storage, sperm membrane integrity was negatively correlated with the percentage of sperm that generated NO (*p* ≤ 0.05) in control samples (Table 3). The negative correlation between membrane integrity and the percentage of sperm that generated NO (*p* ≤ 0.05) was noted after 24 h of semen preservation (Table 3). In turn, after 48 h of storage correlations between membrane integrity and sperm motility were demonstrated (as described above the Table 2).

As soon as 2 h after semen preservation, mitochondrial membrane potential was positively correlated with SOD activity in all samples, except Zn25NPs (Table 4). The next 24 and 48 h demonstrated the presence of significant correlations between MMP and SOD activity (Table 4). The strongest correlations (*p* ≤ 0.05) were found in samples with the addition of Mn25NPs. Interestingly, negative moderate (Mn25NPs) and strong (Mn50NPs) correlations were noticed between MMP and nitric oxide production (Table 4).

All correlations between SOD activity and the rest of examined parameters (Table 5), were described previously above the Table 2, Table 3 and Table 4.

## 4. Discussion

Artificial insemination (AI) is vital for successful reproduction in turkeys, which is why effective semen preservation techniques play a very important role in turkey breeding. Insemination with fresh semen produces the best results, but modern techniques can be applied to preserve semen viability for up to 48 h [16]. The quality of preserved semen can be improved by modifying the composition of the medium. Substances with buffering and antioxidant properties have attracted the interest of turkey breeders in recent years [17].

Due to their low toxicity and high bioavailability, NPs can be effectively used to preserve semen. The influence of NPs is determined by the chemical properties of the applied compound (size, surface charge, coating) as well as the specificity of the biological system (animal species, tissue and cell variability) [18]. The biological effects of NPs can be harnessed in assisted reproduction, in particular to enhance the quality of sperm cells in vivo or in vitro [19].

Previous studies have demonstrated that trace elements such as zinc and manganese may modulate reproductive functions, enhance semen quality and/or contribute to sterility [19]. The presence of zinc and/or manganese ions in seminal plasma was positively correlated with an antioxidant potential of this milieu and negatively associated with lipid peroxidation [20]. Zinc influences the motility of spermatozoa by controlling energy utilization in the ATP system, which participates in muscle contraction and the regulation of phospholipid energy reserves. Zinc ions are firmly bound to the surface of sperm cells. High concentrations of zinc are essential for preserving the viability and fertility of buffalo spermatozoa [21]. Halo Jr. et al. [22] demonstrated the significant effects of ZnO nanoparticles on total motility, viability and cell integrity of stored rabbit spermatozoa. However, they also showed that the spermatotoxicity of ZnO is strictly dose and time dependent. The treatment of zinc nanoparticles improves the plasma membrane integrity and mitochondria activity, simultaneously decreasing the level of MDA in bovine sperm subjected to freezing/thawing processes [23]. An interesting survey was performed by Shahin et al. [24], who noticed that ZnONPs added to the semen extender improved progressive motility, vitality and membrane integrity, both in cooled and frozen-thawed dromedary camel epididymal sperm. In the case of cryopreserved semen, zinc also firmly limited the apoptosis degree and improved ultrastructural morphology. The addition of ZnO to semen extenders has also been found to enhance the freezability of rooster spermatozoa. However, the quadratic response functions for most of the evaluated attributes revealed that the optimal ZnO dose was 0.77–1.27 µg/mL, whereas higher doses compromised post-thaw sperm quality indices [25]. According to Chełmońska and Kassner [26], the average content of zinc in turkey seminal plasma does not exceed 0.62 mg/g. They also suggested that zinc might inhibit the activation of proacrosin, which enhances the fertilizing potential of sperm [27]. Zinc has been found to decrease oxygen uptake and the motility of sperm in several invertebrate and vertebrate species, plus it can influence the survival of turkey spermatozoa in sperm storage tubules [28]. Zinc suppressed oxygen uptake by turkey spermatozoa in vitro, but it did not affect sperm fertility [29]. However, high zinc levels in seminal plasma can decrease sperm activity. Spermatozoa are maintained in a relatively quiescent state when zinc is present in seminal plasma, which promotes energy storage before fertilization. In contrast, the absence of zinc in seminal plasma can destabilize DNA, speed up the acrosome reaction and lead to the loss of ATP [30]. The above observations were confirmed in the present study, where the presence of ZnNPs in the medium was directly correlated with sperm motility. Sperm motility decreased significantly already after 2 h of storage, and it continued to decline in successive hours of storage. The presence of ZnNPs induced a simultaneous decrease in MMP and mitochondrial activity. In semen samples preserved with the addition of ZnNPs, spermatozoa were viable but “inactive” and not motile, which confirms that zinc inhibits the motility of turkey spermatozoa by suppressing oxygen uptake. A decrease in the percentage of sperm producing NO was indicative of low mitochondrial activity. Similarly to other animal species, plasma membrane integrity was maintained in samples of turkey semen preserved with both 25 and 50 μM of ZnNPs, but exposure to higher doses probably exerted cytotoxic effects.

Manganese (Mn^2+^), a more potent stimulator of sperm adenylate cyclase than other metal ions (Co^2+^, Cd^2+^, Zn^2+^, Mg^2+^, Ca^2+^) [31], is an allosteric regulator of adenylyl cyclase. Manganese stimulates sperm motility in a time- and dose-dependent manner [9]. High Mn^2+^ concentrations may be harmful in certain cases, but lower doses are generally safe and effective. A decrease in the Mn content of sperm cells can suppress SOD activity [32]. Bansal [33] demonstrated that Mn^2+^ has a beneficial effect on sperm survival during capacitation and the acrosome reaction. Bansal et al. [34] suggested that Mn^+2^ supplementation can improve the quality of bull semen by minimizing ROS production during storage at 4 °C. They postulated that due to its antioxidant effects, Mn^2+^ stabilizes the plasma membrane and, consequently, increases membrane integrity and sperm viability. They also alleged supplementing bull sperm with a safe dose of manganese up to 60 µM NPs, which permits the rise in the intracellular calcium (Ca^2+^) level without decreasing the viability [8]. On the other hand, Cheema et al. [35] showed that 200 μM manganese significantly limits the peroxidation of membrane lipids and the leakage of proteolytic enzymes from spermatozoa, whereas Lapointe et al. [36] ascertained that addition of 100 μM manganese nanoparticles to extended bull semen stimulates their motility for at least 6 h at temperature of 4 °C. The above could be attributed to high mitochondrial membrane potential maintained by sperm preserved with manganese. In a study by Eidan et al. [37], the addition of Mn^2+^ to a Tris extender significantly increased total counts (×10^6^) of motile spermatozoa, improved acrosomal integrity and increased the counts of spermatozoa with normal morphology relative to control groups in different periods of the sperm production cycle. Manganese easily penetrates sperm cells, and maintains and restores the appropriate ion balance inside cells, thus minimizing the adverse effects of sperm preservation procedures [36]. Miriyala et al. [38] demonstrated that a 50% decrease in Mn-SOD activity resulted in greater damage to sperm DNA. These findings were confirmed in the present study. Above all, the motility parameters of turkey sperm were maintained, but not significantly improved after the addition of MnNPs. In this respect, manganese delivered less satisfactory results in turkeys than in other animal species. However, spermatozoa preserved with the addition of MnNPs were characterized by higher membrane integrity and higher mitochondrial activity (*p* ≤ 0.05), in particular during prolonged storage. At each time point of the analysis, semen samples preserved with Mn25NPs and Mn50NPs demonstrated higher SOD activity than the control samples and samples containing zinc. The above could be attributed to the fact that manganese is a component of MnSOD, the key enzyme that protects energy-generating mitochondria against oxidative damage. This observation could also suggest that external Mn^2+^ is required for maintaining Mn-SOD activity in turkey spermatozoa. These findings also indirectly indicate that SOD influences the physiology of turkey sperm.

ZnNPs and MnNPs could exert beneficial effects on the stored spermatozoa, but further research is needed to validate this observation. In the current study, zinc decreased the motility of turkey sperm over time. Sperm motility is partly responsible for paternity efficiency in turkeys [39], and it is regarded as a key indicator of fertility. It has been scientifically proven that motility depends on the level of nitric oxide formation. At a physiologic level NO takes part in the maintenance of motility, whereas at an exceeded level it leads to motility decrease [40]. In this study, sperm motility could be indirectly associated with the amount of produced NO. The presence of ZnNPs in the medium stimulated sperm to produce NO over long periods of time. However, this finding could also be attributed to the toxic effects of NPs. The results of different surveys also suggest that extracellular zinc affects spermatozoa motility, but the final effect (positive and/or negative) clearly depends on species and the dose [41]. Moreover, zinc supplementation promoted the maintenance of plasma membrane integrity, but only at the lower dose (Zn25NPs). Maybe the zinc dose could be further reduced to 1.5 µg/mL (or less), which was found to be the most effective dose in roosters [25]. Further research is also needed to establish an optimal Mn^2+^ dose for the short-term preservation of semen in a liquid state. Currently, there are no specified data concerning ranges of manganese that can be safely added to the ejaculate without exerting any toxic effects on semen quality or other parameters [20]. Turkey spermatozoa began to lose their fertilizing capability when stored at a low temperature for more than several hours [42]. Fertility problems are more likely to occur when semen is stored for more than 24 h [43]. Future research should focus on improving sperm characteristics directly after collection and prolonging the storage of turkey semen with the use of NPs.

## 5. Conclusions

Our study indicated that turkey ejaculates supplemented with zinc nanoparticles (ZnNPs) and stored for 48 h demonstrated a few declined sperm parameters, i.e., total motility, progressive motility and mitochondrial membrane potential. The addition of zinc NPs to ejaculates led to partial preservation of membrane integrity (samples with Zn25NPs addition) and maintenance of superoxide dismutase activity (both ZnNPs). On the other hand, supplementation of preserved ejaculates with manganese nanoparticles (MnNPs) influenced turkey semen in a greater way. Manganese NPs did not influence sperm motility but enhanced membrane integrity, membrane mitochondrial potential and superoxide dismutase activity, especially during prolonged storage. However, the application of the lower dose of manganese (Mn25NPs) produced better results.

## Figures and Tables

**Table 1:** The effect of time (hours) on the motility characteristics of turkey semen (mean ± SEM) preserved in a liquid state at 4 °C with or without (C) the addition of ZnNPs and MnNPs.

Time (h)	Sample	TMOT [%]	PMOT [%]	VAP [µm/s]	VSL [µm/s]	VCL [µm/s]	ALH [µm]	BCF [Hz]	STR [%]	LIN [%}
2	Zn25	32% ± 6%	27% ± 4%	72.41 ± 0.89	59.39 ± 1.21	112.94 ± 2.47	3.91 ± 0.23	18.67 ± 1.58	81% ± 1%	57% ± 2%
	Zn50	35% ± 6%	31% ± 4%	73.41 ± 0.70	62.64 ± 1.34	111.56 ± 3.07	3.86 ± 0.19	20.39 ± 1.56	84% ± 2%	60% ± 2%
	Mn25	44% ± 6%	31% ± 4%	73.40 ± 0.89	59.12 ± 2.09	116.17 ± 2.69	3.73 ± 0.24	20.83 ± 1.24	80% ± 2%	56% ± 2%
	Mn50	43% ± 6%	31% ± 4%	72.91 ± 1.19	59.59 ± 1.57	113.61 ± 3.60	3.76 ± 0.20	21.99 ± 1.52	82% ± 2%	56% ± 4%
	C	47% ± 5%	31% ± 3%	73.62 ± 0.86	61.90 ± 1.64	113.85 ± 2.36	3.94 ± 0.14	18.35 ± 1.28	83% ± 1%	63% ± 2%
24	Zn25	20% ± 5%	14% ± 3%	48.70 ± 8.39 b	41.98 ± 7.31 c	71.87 ± 12.46 b	2.67 ± 0.41 b	12.77 ± 2.42 c	57% ± 10% c	42% ± 7% c
	Zn50	20% ± 5%	17% ± 4%	52.94 ± 8.56 b	47.90 ± 7.82 b	76.61 ± 12.64 b	2.54 ± 0.45 b	12.46 ± 2.71 c	63% ± 10% b	46% ± 8% b
	Mn25	35% ± 5%	22% ± 3%	75.09 ± 1.49 a	63.31 ± 1.94 a	112.40 ± 2.88 a	3.74 ± 0.17 a	20.49 ± 1.48 a	84% ± 2% a	61% ± 2% a
	Mn50	36% ± 6%	23% ± 4%	72.50 ± 0.92 a	60.41 ± 1.08 a	111.71 ± 2.91 a	4.19 ± 0.26 a	18.09 ± 1.34 b	83% ± 1% a	58% ± 2% a
	C	38% ± 6%	25% ± 3%	73.68 ± 0.72 a	61.57 ± 1.27 a	111.33 ± 2.83 a	3.71 ± 0.16 a	18.23 ± 1.35 b	83% ± 2% a	60% ± 2% a
48	Zn25	16% ± 5% b	12% ± 3% b	43.27 ± 9.51 b	37.62 ± 8.37 b	63.70 ± 14.11 b	2.22 ± 0.50 b	11.44 ± 2.76 b	52% ± 11% b	37% ± 8% b
	Zn50	10% ± 3% b	8% ± 3% b	27.18 ± 9.11 c	24.07 ± 8.10 c	38.02 ± 12.73 c	1.24 ± 0.42 c	6.56 ± 2.35 b	33% ± 11% c	24% ± 8% b
	Mn25	41% ± 6% a	30% ± 4% a	74.93 ± 1.23 a	64.80 ± 1.65 a	110.42 ± 2.33 a	3.57 ± 0.16 a	18.15 ± 1.28 a	86% ± 1% a	63% ± 2% a
	Mn50	42% ± 6% a	29% ± 4% a	73.61 ± 0.77 a	61.68 ± 1.07 a	108.19 ± 2.63 a	3.52 ± 0.12 a	19.11 ± 1.10 a	83% ± 1% a	61% ± 2% a
	C	44% ± 4% a	31% ± 3% a	74.59 ± 1.31 a	64.55 ± 2.15 a	106.98 ± 1.97 a	3.41 ± 0.14 a	17.35 ± 1.44 a	86% ± 2% a	64% ± 2% a

* Different letters (a, b, c) indicate statistically significant differences at *p* ≤ 0.05.

**Table 2:** Correlations between total sperm motility and other sperm parameters during the preservation procedure.

Time [h]	Total Sperm Motility	Membrane Integrity	Mitochondrial Potential	Nitric Oxide	SOD Activity
2	Zn25NPs	−0.039	0.071	0.759 *	0.122
	Zn50NPs	−0.252	0.213	0.746 *	−0.003
	Mn25NPs	0.193	−0.004	0.826 *	0.205
	Mn50NPs	0.008	0.286	0.633 *	0.200
	C	−0.435	0.407	0.174	0.301
24	Zn25NPs	−0.032	0.576 *	0.865 *	0.457
	Zn50NPs	0.085	0.738 *	0.726 *	0.476
	Mn25NPs	−0.242	0.636 *	0.578 *	0.666 *
	Mn50NPs	0.080	0.786 *	0.264	0.498
	C	−0.111	0.442	0.403	0.504
48	Zn25NPs	−0.039	0.092	0.591 *	0.220
	Zn50NPs	0.588 *	−0.180	0.618 *	0.406
	Mn25NPs	0.614 *	0.572 *	−0.307	0.653 *
	Mn50NPs	0.476	0.275	−0.317	0.360
	C	0.122	0.528 *	0.083	0.533 *

* Statistically significant correlations are marked with an asterisk (*p* ≤ 0.05).

**Table 3:** Correlations between membrane integrity and other sperm parameters during the preservation procedure.

Time [h]	Membrane Integrity	Total Sperm Motility	Mitochondrial Potential	Nitric Oxide	SOD Activity
2	Zn25NPs	−0.039	−0.227	0.058	−0.306
	Zn50NPs	−0.252	0.005	−0.056	−0.137
	Mn25NPs	0.193	0.096	0.210	0.144
	Mn50NPs	0.008	−0.300	0.312	−0.384
	C	−0.435	−0.148	−0.702 *	−0.036
24	Zn25NPs	−0.032	0.169	−0.088	−0.106
	Zn50NPs	0.085	0.437	−0.040	0.157
	Mn25NPs	−0.242	−0.036	−0.333	−0.108
	Mn50NPs	0.080	0.140	0.217	0.032
	C	−0.111	−0.009	−0.338	−0.152
48	Zn25NPs	−0.039	−0.348	0.166	0.167
	Zn50NPs	0.588 *	−0.161	0.229	0.205
	Mn25NPs	0.614 *	0.045	0.136	0.318
	Mn50NPs	0.476	0.227	0.132	0.066
	C	0.122	−0.244	0.399	−0.404

* Statistically significant correlations are marked with an asterisk (*p* ≤ 0.05).

**Table 4:** Correlations between membrane mitochondrial potential and other sperm parameters during the preservation procedure.

Time [h]	Mitochondrial Potential	Total Sperm Motility	Membrane Integrity	Nitric Oxide	SOD Activity
2	Zn25NPs	0.071	−0.227	−0.051	0.386
	Zn50NPs	0.213	0.005	−0.098	0.677 *
	Mn25NPs	−0.004	0.096	−0.084	0.521 *
	Mn50NPs	0.286	−0.300	−0.024	0.631 *
	C	0.407	−0.148	−0.306	0.711 *
24	Zn25NPs	0.576 *	0.169	0.402	0.402
	Zn50NPs	0.738 *	0.437	0.381	0.651 *
	Mn25NPs	0.636 *	−0.036	0.285	0.783 *
	Mn50NPs	0.786 *	0.140	−0.093	0.595 *
	C	0.442	−0.009	−0.413	0.706 *
48	Zn25NPs	0.092	−0.348	−0.463	0.195
	Zn50NPs	−0.180	−0.161	−0.189	0.220
	Mn25NPs	0.572 *	0.045	−0.579 *	0.805 *
	Mn50NPs	0.275	0.227	−0.720 *	0.704 *
	C	0.528 *	−0.244	−0.298	0.743 *

* Statistically significant correlations are marked with an asterisk (*p* ≤ 0.05).

**Table 5:** Correlations between SOD activity and other sperm parameters during the preservation procedure.

Time [h]	SOD Activity	Total Sperm Motility	Membrane Integrity	Mitochondrial Potential	Nitric Oxide
2	Zn25NPs	0.122	−0.306	0.386	−0.042
	Zn50NPs	−0.003	−0.137	0.677 *	−0.210
	Mn25NPs	0.205	0.144	0.521 *	0.233
	Mn50NPs	0.196	−0.384	0.631 *	−0.259
	C	0.301	−0.036	0.711 *	−0.233
24	Zn25NPs	0.457	−0.106	0.402	0.218
	Zn50NPs	0.476	0.158	0.651 *	0.195
	Mn25NPs	0.666 *	−0.108	0.683 *	0.049
	Mn50NPs	0.498	0.033	0.595 *	−0.289
	C	0.504	−0.152	0.706 *	−0.191
48	Zn25NPs	0.220	0.167	0.195	0.053
	Zn50NPs	0.406	0.205	0.220	0.217
	Mn25NPs	0.653 *	0.318	0.805 *	−0.567 *
	Mn50NPs	0.360	0.066	0.704 *	−0.834 *
	C	0.533 *	−0.404	0.743 *	−0.273

* Statistically significant correlations are marked with an asterisk (*p* ≤ 0.05).

## Data Availability

The data presented in this study are available upon request from the corresponding author.
